# Pre-pregnancy body mass index (BMI) and maternal gestational weight gain are positively associated with birth outcomes in rural Malawi

**DOI:** 10.1371/journal.pone.0206035

**Published:** 2018-10-23

**Authors:** Austrida Gondwe, Per Ashorn, Ulla Ashorn, Kathryn G. Dewey, Kenneth Maleta, Minyanga Nkhoma, John Mbotwa, Josh M. Jorgensen

**Affiliations:** 1 University of Tampere, Faculty of Medicine and Life Sciences, Centre for Child Health Research, Tampere, Finland; 2 Tampere University Hospital, Department of Paediatrics, Tampere, Finland; 3 Department of Nutrition, University of California Davis, Davis, California, United States of America; 4 University of Malawi College of Medicine, School of Public Health and Family Medicine, Blantyre, Malawi; TNO, NETHERLANDS

## Abstract

**Background:**

Whereas poor maternal nutritional status before and during pregnancy is widely associated with adverse birth outcomes, studies quantifying this association in low income countries are scarce. We examined whether maternal pre-pregnancy body mass index (BMI) and weight gain during pregnancy are associated with birth outcomes in rural Malawi.

**Methods:**

We analyzed the associations between pre-pregnancy BMI and average weekly gestational weight gain (WWG) and birth outcomes [duration of gestation, birth weight, length-for-age z-score (LAZ), and head circumference-for-age z-score (HCZ)]. We also determined whether women with low or high pre-pregnancy BMI or women with inadequate or excessive WWG were at increased risk of adverse birth outcomes.

**Results:**

The analyses included 1287 women with a mean BMI of 21.8 kg/m^2^, of whom 5.9% were underweight (< 18.5 kg/m^2^), 10.9% were overweight (≥ 25 kg/m^2^), 71.8% had low WWG [below the lower limit of the Institute of Medicine (IOM) recommendation], and 5.2% had high WWG (above IOM recommendation). In adjusted models, pre-pregnancy BMI was not associated with duration of pregnancy (p = 0.926), but was positively associated with birth weight and HCZ (<0.001 and p = 0.003, respectively). WWG was positively associated with duration of gestation (p = 0.031), birth weight (p<0.001), LAZ (p<0.001), and HCZ (p<0.001). Compared to normal weight women, underweight women were at increased risk of having stunted infants (p = 0.029). Women with low WWG were at increased risk of having infants with low birth weight (p = 0.006) and small head circumference (p = 0.024) compared to those with normal weight gain. Those with high BMI or high WWG were not at increased risk of adverse birth outcomes.

**Conclusions:**

WWG is an important predictor of birth outcomes in rural Malawi. The high prevalence of inadequate WWG compared to low pre-pregnancy BMI highlights the need to investigate causes of inadequate weight gain in this region.

## Introduction

The nutritional status of a woman before and during pregnancy is important for a healthy pregnancy outcome [1]. Maternal malnutrition may play a key role with regard to poor fetal growth including low birth weight (LBW), short- and long-term infant morbidity and mortality, and long term, potentially irreversible cognitive, motor and health impairments [2,3]. Pregnant women in sub-Saharan Africa are at a particular nutritional risk as a result of poverty, food insecurity, political and economic instabilities, frequent infections and frequent pregnancies [4].

The effects of pre-pregnancy anthropometric status (estimated by maternal BMI) are well documented [4–6]. In low income countries (LICs), maternal underweight in early pregnancy is a leading risk factor for adverse birth outcomes, including LBW, preterm birth, small for gestational age (SGA), and stillbirth [5,6]. There is some evidence suggesting that high BMI prior to pregnancy is associated with an increased risk of preeclampsia, gestational diabetes, caesarean section, postpartum haemorrhage, and fetal macrosomia [7]. Additionally, entering pregnancy with high pre-pregnancy BMI increases the risk for pregnancy complications and adverse outcomes for both mother and infant [8–10].

Maternal gestational weight gain (GWG) has also been widely studied as an independent predictor of adverse pregnancy outcomes [11,12]. Multiple studies from middle and high income countries have found that women with inadequate GWG were at a higher risk of giving birth to LBW and preterm infants [5,12–16]. Women in LICs generally have lower weight and GWG than those in high income countries [17]. More than 95% of LBW infants are born in LICs—sub-Saharan Africa alone has a 15% incidence of LBW [18]. In Malawi, the incidence of LBW is 12% [19]. Additionally, Malawi is one of the poorest countries in sub–Saharan Africa with approximately 80% of the population living in rural communities that are faced with various health challenges including malnutrition [19]. However, recent reports show an increase in obesity/overweight from 9% in 1992 to 21% in 2015–16 among urban Malawian women of reproductive age (15–45 years) [19,20]. Studies that have examined the impact of pre-pregnancy BMI (either low or high) or inadequate or excessive GWG on birth outcomes in Malawi are lacking. In the present study, we tested the hypothesis that maternal pre-pregnancy BMI and weight gain during pregnancy are positively associated with birth outcomes (duration of pregnancy and size of the newborn including weight, length and head circumference). We also aimed to examine the impact of pre-pregnancy BMI (either underweight or overweight) and average weekly gestational weight gain (WWG;(inadequate or excessive) on risk of adverse birth outcomes (preterm birth, stunting, LBW, small head circumference and SGA). In an exploratory analysis, we examined whether a lipid-based nutrient supplement or a multiple micronutrient supplement consumed during pregnancy modulated the associations between pre-pregnancy BMI and birth outcomes compared to an iron-folic acid supplement.

## Materials and methods

### Study design and setting

This was a prospective cohort study, nested within the International Lipid-Based Nutrient Supplements (iLiNS) Project DYAD trial in Malawi (iLiNS-DYAD-M), which was a randomised controlled trial that was carried out in Mangochi District. Mangochi district is a semi-urban, semi-rural area of southern Malawi with subsistence farming and fishing as the main sources of income. Participants were recruited between 14 and 20 gestation weeks, seen again at 32 and 36 gestation weeks, and soon after birth, to determine weight gain during pregnancy and birth outcomes.

Participants were recruited from 1 district hospital (Mangochi), 1 semi-private hospital (Malindi) and 2 public health centres (Lungwena and Namwera) from February 2011 to August 2012. The inclusion criteria were being a permanent resident of the catchment areas, not more than 20 weeks gestational age, identified through antenatal clinics and signed or thumb-printed informed consent. The exclusion criteria were being less than 15 years of age, chronic medical conditions requiring frequent medical attention, history of allergies, evident pregnancy complications, earlier participation in the same trial or concurrent participation in any other clinical trial. Participants in the trial were randomized into three intervention groups. The women consumed from ≤ 20 gestation weeks until delivery either one daily iron and folic acid (IFA) capsule; one capsule with 18 micronutrients (MMN); or one 20 g sachet of lipid-based nutrient supplement (LNS) containing 118 kcal, protein, carbohydrates, essential fatty acids, and 22 micronutrients [21] (**S1 Table**). The intervention had a limited impact on birth outcomes [22]. The trial was performed according to Good Clinical Practice guidelines and the ethical standards of the Helsinki Declaration. The study (Trial registration: www.clinicaltrials.gov, trial identification NCT01239693) was conducted under approval of College of Medicine Research Ethics Committee (COMREC), University of Malawi, and the Ethics Committee of Pirkanmaa Hospital District, Finland.

The study nurses determined the women’s gestational age through obstetric ultrasound assessment. We obtained participants’ background information and details of their home location at enrolment day. To examine maternal BMI and maternal weight gain during pregnancy, the study staff completed clinic anthropometric measurements at enrolment (≤ 20 weeks), 32 weeks and 36 weeks gestational age. The anthropometrists assessed the weight and height of the mother using a high quality scale (SECA 874 flat scale, Seca GmbH & Co., Hamburg, Germany) and stadiometer (Harpenden stadiometer, Holtain Limited, Crosswell, Crymych, UK). We assessed infant weight using an electronic infant weighing scale (SECA 381 baby scale, Seca GmbH & Co.), infant length using length boards (Harpenden Infantometer, Holtain Limited) and head circumference with non-stretchable plastic insertion tapes (Shorrtape, Weigh and Measure, LLC, Olney, MD, USA).

### Definitions

Maternal pre-pregnancy BMI was categorised into underweight (<18.5 kg/m^2^), normal (18.5–24.9 kg/m^2^), and overweight (≥ 25 kg/m^2^), according to the classification by the World Health Organisation (WHO) [23]. Average WWG was defined as the average weight gained per week during pregnancy based on Institute of Medicine (IOM) for weight gain during pregnancy [24]. The IOM guidelines were developed to minimise the negative health consequences for the mother and fetus of inadequate or excessive gain. The guidelines recommend that underweight women gain more weight, and overweight women gain less weight, compared to women within the “normal” weight category at the time of conception [25]. Inadequate weight gain was defined as average weekly weight gained below the IOM guidelines for average weekly weight gain during pregnancy and excess weight gain was defined as average weekly weight gained above the IOM guidelines for weight gain during pregnancy [25]. The cut-off for inadequate rate of WWG was the lower limit of the IOM’s recommended range of average weekly weight gain during pregnancy, which takes the pre-pregnancy BMI into consideration [25]. Preterm birth was defined as < 37 weeks of gestation, newborn underweight as < 2500 g, stunting as newborn length for age z-score (LAZ) < -2, small head circumference as newborn head circumference z-score (HCZ) < −2, and small for gestational age (SGA) as weight <10^th^ percentile for gestational age and sex using the INTERGROWTH -21^st^ standards [26]. We used the WHO Child Growth Standards to calculate the weight-for-age, length-for-age, and head circumference-for-age z-scores [27].

### Statistical analysis

Since pre-pregnancy BMI was not available for study participants, we used regression modelling to create a proxy for pre-pregnancy BMI. We created a regression curve of maternal BMI against gestational age at enrolment and determined that 13.7 gestational weeks (gw) was the lower limit of the curve at which the 95% CI fit closely to the regression curve. We therefore used the estimated BMI at 13.7 gw as the proxy for pre-pregnancy BMI and assumed that minimal change in BMI occurred between pre-pregnancy and 13.7 gw, based on IOM assumptions of weight gain in the first trimester (0.5–2.0 kg) [25]. WWG as a continuous variable was estimated using a mixed modelling method. This method allowed us to calculate the average weekly weight gain for all women from whom weight measurement was performed at each clinic visit (enrolment, 32 gw, or 36 gw), and impute weekly weight gain for those from whom one or two weight measurements were missing.

We used analysis of variance (ANOVA) for continuous variables and Fisher’s exact test for categorical variables to examine differences between those included and excluded from the analyses of associations between maternal weight gain and pre-pregnancy BMI and birth outcomes. We used linear regression models to examine the associations between pre-pregnancy BMI, weekly weight gain and continuous birth outcome variables (duration of gestation, birth weight, LAZ, and HCZ). We used logistic regression to examine the association between categorical and continuous variables. We also examined the interaction between pre-pregnancy BMI and weekly weight gain (as both continuous and categorical variables) with regard to maternal birth outcomes. Linear regression coefficients are presented as standardized coefficients, which are the number of standard deviations a dependent variable will change, per standard deviation increase in the predictor variable. We used standardized coefficients in order to assess the strength of association between the predictor and birth outcome in a given model in comparison with the association between another predictor and that same birth outcome in a different model. The use of standardized coefficients allows for such comparisons between predictor variables for the same outcome, even though the units of the predictor variables differ (i.e. BMI vs. weekly weight gain). We computed standardized coefficients from the regression analysis using standardized dependent and independent variables. We used Poisson regression models to estimate the relative risk for dichotomous birth outcomes (preterm birth, LBW, stunting, SGA, and small head circumference) by categorical predictors of pre-pregnancy BMI (<18.5 vs. between 18.5 and 24.9, and ≥ 25.0 vs. between 18.5 and ≥ 25.0) and average WWG. Potential confounders such as maternal age, maternal height, haemoglobin (HB) at enrolment, HIV status, season of enrolment, site of enrolment, primiparity, marital status and education years were selected *a priori* based on their reported associations with birth outcomes and examined as covariates. Those that were associated with the birth outcomes (p<0.10) in bivariate analysis were included in the adjusted models. All hypothesis tests were two-sided and P values <0.05 were considered statistically significant. Data analyses were performed using STATA version 12.1 (Stata Corp, College Station, TX, USA) and SAS version 9.3 software package (SAS Institute Inc., Cary, NC, USA).

## Results

A total of 1391 participants were enrolled in the iLiNS-DYAD-M study between February 2011 and August 2012. Of the 1391 participants enrolled, we had complete information on height and weight for 1382 participants (99.4%). After excluding 12 women with twin pregnancies, BMI at enrolment was available for 1370 (98.5%) participants. Data on the duration of pregnancy were available for 1287 (93.9% of those from whom BMI data were available) and birth weight, length, and head circumference were available for 79.7%, 79.0%, and 79.1% of those from whom BMI data were available, respectively. Birth outcome data were not available for approximately 20% of newborns of the enrolled participants because some died, some moved out of the area, some weren’t found at the time of measurements, and some newborns didn’t cooperate (stay still) for the weighing or measuring.

The mean (SD) pre-pregnancy BMI of all participants was 21.8 (2.7) kg/m^2^. The proportion of women who were underweight (BMI < 18.5 kg/m^2^) was 5.9%, while the prevalence of overweight (BMI ≥ 25.0 kg/m^2^) was 10.9%. The proportion of women who had low WWG (< IOM recommendation) was 71.8%, while the prevalence of high WWG was 5.2%. There were differences in baseline characteristics of women included and excluded from the analyses of associations between BMI and weight gain and birth outcomes (**Table 1**). Those who were included in the analyses were older, less educated, of lower socio-economic status, more likely to be primiparous, and less likely to have had low WWG compared to women who were excluded from the analyses.

**Table 1:** Baseline characteristics of the participants who were included and excluded from the analyses of associations between pre-pregnancy BMI and gestational weight gain and birth outcomes (n = 1379) in Malawi.

Characteristic	Included (n = 1107)	Excluded (n = 272)	*P* value^a^
Mean (SD) body-mass index, BMI (kg/m2)	22.1 (2.8)	22.4 (2.9)	0.126
Mean (SD) maternal age, years	25.1 (6.1)	24.2 (6.6)	0.025
Mean (SD) maternal education, completed years at school	3.9 (3.4)	4.5 (3.7)	0.049
Mean (SD) socio-economic score	-0.05 (0.9)	0.30 (1.1)	<0.001
Proportion of primiparous women	29.6%	20.1%	<0.001
Proportion of women with a low BMI (< 18.5 kg/m^2^)	4.1%	5.7%	0.367
Proportion of women with low weekly weight gain (< IOM recommendation)	70.2%	79.9%	0.008
Proportion of women with a positive HIV test	12.2%	13.9%	0.591
Number (%) of women with a positive malaria test (RDT)	22.4%	23.5%	0.749

^a^ *P* value obtained from ANOVA (comparison of means) or Fishers exact test (comparison of proportions)

### Associations between pre-pregnancy BMI and weekly GWG and duration of pregnancy and newborn size

Pre-pregnancy BMI was positively associated with birth weight and HCZ in both unadjusted (β = 0.11, *p* = 0.001and β = 0.09, *p* = 0.003, respectively) and adjusted models (β = 0.11, *p* = 0.001 and β = 0.09, *p* = 0.003, respectively) **(Table 2)**. No statistical associations were seen between pre-pregnancy BMI and pregnancy duration in either unadjusted (β = -0.01, *p* = 0.686) or adjusted models (β = -0.003, *p* = 0.926). However, in unadjusted models pre-pregnancy BMI was associated with LAZ (β = -0.065, *p* = 0.040) and HCZ (β = 0.09, *p* = <0.003) but the association with LAZ was no longer significant after adjusting for covariates (β = -0.056, *p* = 0.067). WWG was significantly associated with pregnancy duration, birthweight, LAZ and HCZ in both unadjusted (β = 0.08, *p* = 0.002; β = 0.22, *p* = 0.001; β = 0.19, *p* = 0.001 and β = 0.15, *p* = 0.001) and adjusted models (β = 0.06, *p* = 0.031; β = 0.20, *p* = 0.001; β *=* 0.13, *p* = 0.001; and β *=* 0.15, *p* = 0.001, respectively). There were no significant interactions between pre-pregnancy BMI and WWG for any of the birth outcomes.

**Table 2:** Associations between maternal pre-pregnancy body-mass index (BMI) and average weekly gestational weight gain and duration of pregnancy and newborn size in rural Malawi.

Outcome	Pre-pregnancy BMI (kg/m^2^)				Weekly weight gain (g/wk)			
	Unadjusted		Adjusted		Unadjusted		Adjusted	
	Regression co-efficient (SE)^a^	P-value^b^	Regression co-efficient (SE)^a^	P-value^b^^,^^c^	Regression co-efficient (SE)^a^	P-value^b^	Regression co-efficient (SE)^a^	P-value^b^^,^^c^
Pregnancy duration	-0.01 (0.03)	0.686	-0.003 (0.03)	0.926	0.08 (0.03)	<0.002	0.06 (0.03)	0.031
Birthweight	0.11(0.03)	<0.001	0.11(0.03)	<0.001	0.22 (0.03)	<0.001	0.20 (0.03)	<0.001
Newborn length-for-age z-score	0.065 (0.03)	0.040	0.056 (0.03)	0.067	0.19 (0.03)	<0.001	0.13 (0.03)	<0.001
Newborn head circumference z-score	0.09(0.03)	<0.003	0.09 (0.03)	0.003	0.15 (0.03)	<0.001	0.15 (0.03)	<0.001

^a^ Standardized coefficient with standardized standard errors. Standardized coefficients are the number of standard deviations the outcome variable changes per standard deviation change in the predictor variable.

^b^ P-values were determined by linear regression models.

^c^Models were adjusted for covariates found in bivariate analysis to be associated with the birth outcome (P<0.10). Model for pregnancy duration was adjusted for gestational age at enrollment, parity, maternal height and HIV status. Model for birthweight was adjusted for number of previous pregnancies, HIV status, primparity, site of enrollment, season of enrollment, and maternal. Model for length age z score (LAZ) was adjusted for number of previous pregnancies, HIV status, child sex, maternal height, parity, and maternal age. Model for head circumference was adjusted for number of previous pregnancies, maternal height, parity and site of enrollment. Models for the associations between weekly weight gain and duration of pregnancy, birthweight, LAZ, and head circumference were also adjusted for maternal pre-pregnancy BMI.

### Associations between low or high BMI and birth outcomes

The associations between low and high BMI and birth outcomes are presented in **Table 3** and **Table 4**. Mean duration of pregnancy and LAZ were not different between women who were underweight compared to women of normal weight [mean difference (95% CI): -0.22 wk (-0.93 to 0.50), *p* = 0.536; and -0.25 z-score (-0.08 to 0.57), *p* = 0.137, respectively). Similarly, no significant differences were noted in either average birth weight [mean difference (95% CI): 66 g (-64 to 196 g), *p* = 0.320] or HCZ [mean difference (95% CI): 0.20 z-score (-0.16 to 0.48), *p* = 0.333] among infants born to underweight women and normal weight women (**Table 3**). However, we found that infants born to underweight women were at a greater risk of stunting [RR (95% CI): 1.6 (1.0 to 2.5), *p* = 0.029] and being SGA [RR (95% CI): 1.5 (1.2 to 2.0), *p* = 0.002] but no significant risk was noted for small head circumference [RR (95% CI): 0.9 (0.3 to 2.8), *p* = 0.827] when compared to infants born to normal weight women (**Table 4**). We found no increase or decrease in risk of stunting, SGA, or small head circumference when comparing infants of overweight and normal weight women in adjusted models [RR, (95% CI): 0.9 (0.5 to 1.5), *p* = 0.634; 0.7 (0.5 to1.0), *p* = 0.075; and 0.7 (0.2 to 2.4), *p* = 0.620, respectively].

**Table 3:** Continuous birth outcomes among normal weight, underweight, and overweight women in rural Malawi.

Outcome	Maternal nutritional status before pregnancy (based on body-mass-index BMI)			Comparison between underweight and normal weight participants				Comparison between overweight and normal weight participants			
	Normal BMI (18.5–25.0 kg/m^2^)	Underweight (BMI < 18.5 kg/m^2^)	Overweight (BMI ≥25.0 kg/m^2^)	Unadjusted		Adjusted		Unadjusted		Adjusted	
				Mean Difference (95% CI)	p-value^a^	Mean Difference (95% CI)	p-value^a^^,^^b^	Mean Difference (95% CI)	p-value^a^	Mean Difference (95% CI)	p-value^a^^,^^b^
Mean (SD)^c^ duration of pregnancy, weeks	39.1 (3.0) *n = 1058*	39.5 (2.2) *n = 71*	39.2 (2.7) *n = 158*	-0.46 (-1.17 to 0.25)	0.200	-0.22 (-0.93 to 0.50)	0.536	-0.16 (-0.34 to 0.66)	0.527	- 0.08 (-0.57 to 0.42)	0.761
Mean (SD) birthweight (grams)	2973 (446.8) *n = 941*	2939 (446.9) *n = 62*	3012 (439.9) *n = 133*	134 (20 to 249)	0.022	66 (-64 to 196)	0.320	-39 (-120 to -42)	0.350	108.0 (- 9 to 225)	0.070
Mean (SD) length for age z-score	-1.00 (1.11) *n = 893*	-1.21 (1.09) *n = 60*	-0.87 (1.10) *n = 129*	0.21 (-0.08 to 0.50)	0.163	0.25 (-0.08 to 0.57)	0.137	- 0.13 (-0.34 to 0.07)	0.211	0.02 (-0.27 to 0.31)	0.898
Mean (SD) head circumference z-score	*-0*.*14* *(1*.*08)* *n = 894*	-0.47 (0.92) *n = 61*	-0.06 (1.17) *n = 129*	0.34 (0.06 to 0.62)	0.018	0.20 (-0.16 to 0.48)	0.333	-0.08 (-0.28 to 0.12)	0.429	0.21 (-0.08 to 0.51)	0.160

^a^ P-values obtained from Poisson regression models.

^b^ Models were adjusted for covariates found in bivariate analysis to be associated with the birth outcome (P<0.10). Model for gestational age at delivery was adjusted for gestational age at enrollment, parity, maternal height and HIV status. Model for birthweight was adjusted for number of previous pregnancies, HIV status, primparity, site of enrollment, season of enrollment, maternal age and gestational age at enrolment. Model for length for age-z score was adjusted for number of previous pregnancies, HIV status, child sex, maternal height, parity, maternal age, and gestational age at enrolment. Model for head circumference was adjusted for number of previous pregnancies, maternal height, parity and site of enrollment.

^c^ SD: standard deviation

**Table 4:** The prevalence of adverse birth outcomes among normal weight, underweight, and overweight women in rural Malawi.

Outcome	Maternal nutritional status before pregnancy (based on body-mass-index BMI)			Comparison between underweight and normal weight participants				Comparison between overweight and normal weight participants			
				Unadjusted		Adjusted		Unadjusted		Adjusted	
	Normal BMI(18.5–25.0 kg/m^2^)	Underweight (BMI < 18.5 kg/m^2^)	Overweight (BMI ≥25.0 kg/m^2^)	RR (95% CI)	P-value^a^	RR (95% CI)	p-value^a^^,^^b^	RR (95% CI)	p-value^a^	RR (95% CI)	p-value^a^^,^^b^
Incidence of preterm (GA<37wk)	110/1060 (10.4%)	5/93 (5.4%)	14/134 (10.5%)	0.5 (0.2 to 1.2)	0.139	0.6 (0.2 to 1.4)	0.202	1.0 (0.6 to 1.7)	0.980	1.0 (0.6 to 1.7)	0.908
Incidence of low birth weight (<2500 g)	118/942 (12.5%)	15/81 (18.5%)	12/113 (10.6%)	1.5 (0.9 to 2.4)	0.115	1.5 (0.9 to 2.5)	0.100	0.8 (0.5 to 1.5)	0.560	0.8 (0.5 to 1.4)	0.464
Prevalence of stunting (LAZ<-2)	140/894 (15.7%)	20/80 (25%)	13/108 (12.0%)	1.6 (1.1 to 2.4)	0.024	1.6 (1.0 to 2.5)	0.029	0.8 (0.5 to 1.3)	0.332	0.9 (0.5 to 1.5)	0.634
Prevalence of small for gestational age (SGA)^c^	279/942 (29.6%)	34/81 (42.0%)	22/113 (19.5%)	1.4 (1.1 to 1.9)	0.013	1.5 (1.2to 2.0)	0.002	0.7 (0.4 to 1.0)	0.034	0.7 (0.5 to 1.0)	0.075
Prevalence of small head circumference (HCZ<-2)	37/894 (4.1%)	3/81 (3.7%)	3/109 (2.8%)	0.9 (0.3 to 2.8)	0.850	0.9 (0.3 to 2.8)	0.827	0.7 (0.2 to 2.1)	0.491	0.7 (0.2 to 2.4)	0.620

^a^ P-values obtained from Poisson regression models.

^b^ Models were adjusted for covariates found in bivariate analysis to be associated with the birth outcome (P<0.10). Model for preterm birth was adjusted for gestational age at enrollment, HIV status, primiparity, and site of enrollment. Model for LBW was adjusted for child sex, HIV status, maternal height, primiparity, household food insecurity score, and site of enrollment. Model for newborn stunting was adjusted for HIV status, maternal age, maternal height, primiparity, and site of enrollment. Model for SGA was adjusted for child sex, gestational age at enrollment, HIV status, maternal age, maternal height, primiparity, season of enrollment, and site of enrollment. Model for small head circumference was adjusted for maternal age, maternal height, primiparity, season of enrollment, and site of enrollment

^c^ Defined as having birth weight <10th percentile for infants of the same gestational age from INTERGROWTH-21st standard

GA: Gestational age

### Associations between low or high WWG and birth outcomes

Compared to women whose WWG was normal (within IOM recommendations), women with low WWG (below the IOM recommendations) in adjusted models had a shorter average duration of pregnancy [mean difference (95% CI): 0.56 wk (0.17 to 0.95), *p* = 0.005]; lower average infant birth weight [mean difference (95% CI): 142 g (80 to 204), *p* = 0.001]; LAZ [mean difference (95% CI): 0.26 z-score (0.11 to 0.41), *p* = 0.001]; and head circumference z-score [mean difference (95% CI): 0.33 z-score (0.18 to 0.49), *p* = 0.001] (**Table 5**). Similarly, women with low WWG were at a greater risk of having an infant with LBW, SGA and small head circumference in adjusted models [RR (95% CI): 2.0 (1.2 to 3.2), *p* = 0.006; 1.4 (1.0 to 1.8), *p* = 0.037; and 3.4 (1.2 to 9.7), p = 0.024, respectively], but not significantly more likely to have a stunted infant or give birth preterm [RR (95% CI): 1.1(0.7 to 1.6), *p* = 0.681; and 1.5 (0.9 to 2.3), *p* = 0.124, respectively] (**Table 6**). There were no differences in birth outcomes or risk of adverse birth outcomes among women with high WWG (above the IOM recommendations) compared to women with normal WWG.

**Table 5:** Continuous birth outcomes among women with normal, low, or high average weekly gestational weight gain in rural Malawi.

Outcome				Comparison between women with low and normal weight gain				Comparison between women with high and normal weight gain			
	Maternal weight during pregnancy			Unadjusted		Adjusted		Unadjusted		Adjusted	
	Normal weight gain within IOM recommendations	Low (below IOM recommendations	High (above IOM recommendations)	Difference in means (95% CI)	P-value^a^	Difference in means (95% CI)	P-value^a^^,^^b^	Difference in means (95% CI)	P-value^a^	Difference in means (95% CI)	P-value^a^^,^^b^
Mean (SD)^c^ duration of pregnancy, weeks	39.63 (2.18) *n = 302*	38.89 (3.18) *n = 916*	40.04 (1.61) *n = 69*	0.74 (0.35 to 1.12)	<0.002	0.56 (0.17 to 0.95)	0.005	-0.41 (-0.91 to 0.13)	0.138	-0.45 (-0.99 to 0.08)	0.099
Mean (SD) birthweight (grams)	3098 (451) *n = 278*	2908 (440) *n = 797*	3203 (311) *n = 61*	190 (130 to 251)	<0.001	142 (80 to 204)	<0.001	-105 (-224 to 15)	0.086	-88.51 (-212 to 35)	0.158
Mean (SD) length for age z score	-0.72 (1.04) *n = 257*	-1.13 (1.12) *n = 767*	-0.47 (0.83) *n = 58*	0.41 (0.25 to 0.56)	<0.001	0.26 (0.11 to 0.41)	<0.001	- 0.25 (-0.54 to 0.03)	0.084	-0.24 (-0.54 to 0.05)	0.102
Mean (SD) head circumference headz	0.07 (0.95) *n = 261*	0.26 (1.12) *n = 765*	0.34 (0.91) *n = 58*	0.31 (0.2 to 0.5)	<0.001	0.33 (0.18 to 0.49)	<0.001	-0.26 (-0.53 to 0.01)	0.055	-0.26 (-0.49 to 0.07)	0.139

^a^ P-values obtained from Poisson regression models.

^b^ Models were adjusted for covariates found in bivariate analysis to be associated with the birth outcome (P<0.10). Model for gestational age at delivery was adjusted for gestational age at enrollment, parity, maternal height and HIV status. Model for birthweight was adjusted for number of previous pregnancies, HIV status, primparity, site of enrollment, season of enrollment, maternal age and maternal BMI. Model for length for age-z score was adjusted for number of previous pregnancies, HIV status, child sex, maternal height, parity and maternal age. Model for head circumference was adjusted for number of previous pregnancies, maternal height, parity and site of enrollment.

^c^ SD: standard deviation

**Table 6:** The prevalence of adverse birth outcomes among women with normal, low, or high average weekly gestational weight gain in rural Malawi.

Outcome				Comparison between women with low and normal weight gain				Comparison between women with high and normal weight gain			
	Maternal weight gain during pregnancy			Unadjusted		Adjusted		Unadjusted		Adjusted	
	Normal weight gain within IOM^d^ recommendations	Low (below IOM recommendations	High (above IOM recommendations)	RR (95% CI)	P-value^a^	RR (95% CI)	P-value^a^^,^^b^	RR (95% CI)	P-value^a^	RR (95% CI)	P-value^a^^,^^b^
Incidence of preterm birth (GA <37 weeks)	21/302 (7.0%)	106/916 (11.6%)	2/69 (2.9%)	1.7 (1.0 to 2.7)	0.033	1.5 (0.9 to 2.3)	0.124	0.4 (0.1 to 1.8)	0.237	0.4 (0.1 to 1.6)	0.178
Incidence of (low birth weight) LBW (<2,500 g)	21/278 (7.6%)	124/797 (15.6%)	0/61 (0.0%)	2.1 (1.3 to 3.3)	0.002	2.0 (1.2 to 3.2)	0.006	N/A	N/A	N/A	N/A
Prevalence of newborn stunting (LAZ <-2)	35/257 (13.6%)	138/767 (18.0%)	0/58 (0.0%)	1.3 (0.9 to 1.9)	0.141	1.1 (0.7 to 1.6)	0.681	N/A	N/A	N/A	N/A
Incidence of small for gestational age (SGA)^c^	64/278 (23.0%)	265/797 (24.7%)	6/61 (9.8%)	1.4 (1.1 to 1.9)	0.008	1.4 (1.0 to 1.8)	0.037	0.4 (0.2 to 1.0)	0.046	0.5 (0.2 to 1.2)	0.120
Prevalence of small head circumference (HCZ <-2)	4/261 (1.5%)	39/765 (5.1%)	0/58 (0.0%)	3.3 (1.2 to 9.3)	0.022	3.4 (1.2 to 9.7)	0.024	N/A	N/A	N/A	N/A

^a^ P-values for incidence of preterm birth and prevalence of small for gestational age newborns were obtained from Poisson regression models. P-values for unadjusted incidence of LBW, and prevalence of stunting and small head circumference were obtained by Fisher’s exact test, as no `women who gained excess weight gave birth to a LBW or stunted infant or an infant with a small head circumference.

^b^ Models were adjusted for covariates found in bivariate analysis to be associated with the birth outcome (P<0.10). Model for preterm birth was adjusted for gestational age at enrollment, HIV status, primiparity, and site of enrollment. Model for LBW was adjusted for child sex, HIV status, maternal height, primiparity, household food insecurity score, and site of enrollment. Model for newborn stunting was adjusted for HIV status, maternal age, maternal height, primiparity, and site of enrollment. Model for SGA was adjusted for child sex, gestational age at enrollment, HIV status, maternal age, maternal height, primiparity, season of enrollment, and site of enrollment. Model for small head circumference was adjusted for maternal age, maternal height, primiparity, season of enrollment, and site of enrollment

^c^ Defined as having birth weight <10th percentile for infants of the same gestational age from INTERGROWTH-21st standard

^d^ IOM: Institute of Medicine

### Associations between pre-pregnancy BMI and birth outcomes within each intervention group

There were no significant associations between pre-pregnancy BMI and either pregnancy duration or LAZ when stratified by intervention group after adjusting for covariates (**Table 7**). However, the covariate adjusted association between pre-pregnancy BMI and birth weight was significantly positive among all 3 intervention groups (IFA: p = 0.012; MMN: p = 0.007; LNS: p = 0.033). There was a significantly positive association between pre-pregnancy BMI and HCZ only among women in the MMN group (p = 0.019), but not the IFA (p = 0.096) or LNS (p = 0.140) groups.

**Table 7:** Associations between maternal pre-pregnancy body-mass index (BMI) and duration of pregnancy and newborn size within each intervention group.

Outcome	Group	Pre-pregnancy BMI (kg/m^2^)	
		Regression co-efficient (SE)^a^	P-value^b^
Pregnancy duration	IFA	-0.08 (0.05)	0.129
	MMN	0.01 (0.05)	0.816
	LNS	0.03 (0.05)	0.561
Birthweight	IFA	0.14 (0.06)	0.012
	MMN	0.14 (0.05)	0.007
	LNS	0.11 (0.05)	0.033
Newborn length-for-age z-score	IFA	0.08 (0.06)	0.225
	MMN	0.08 (0.05)	0.094
	LNS	0.04 (0.05)	0.364
Newborn head circumference z-score	IFA	0.11 (0.06)	0.096
	MMN	0.13 (0.05)	0.019
	LNS	0.07 (0.05)	0.140

^a^ Standardized coefficient with standardized standard errors. Standardized coefficients are the number of standard deviations the outcome variable changes per standard deviation change in the predictor variable.

^b^ P-values were determined by linear regression models. Models were adjusted for covariates found in bivariate analysis to be associated with the birth outcome (P<0.10). Model for gestational age at delivery was adjusted for gestational age at enrollment, parity, maternal height and HIV status. Model for birthweight was adjusted for number of previous pregnancies, HIV status, primiparity, site of enrollment, season of enrollment, and maternal age. Model for length for age z score was adjusted for number of previous pregnancies, HIV status, child sex, maternal height, parity, and maternal age. Model for head circumference was adjusted for number of previous pregnancies, maternal height, parity and site of enrollment.

Within each intervention group, the risk of preterm birth, low birthweight, stunting, and small head circumference was not different between women with a BMI in the normal range compared to women with either a low or high BMI after adjusting for covariates. The risk of SGA was significantly higher among women with a low compared to normal BMI for women in both the IFA [RR (95% CI): 1.6 (1.01 to 2.4), p = 0.047)] and MMN [RR (95%CI): 1.9 (1.2 to 3.1), p = 0.008] groups, but not among women in the LNS group [RR (95%CI): 1.3 (0.7 to 2.1), p = 0.404]. There were no significant differences in adverse birth outcomes between women with low vs higher BMI within any of the intervention groups (p>0.05 for all) (**Table 8**).

**Table 8:** The prevalence of adverse birth outcomes among normal weight, underweight, and overweight women within each intervention group.

Outcome		Maternal nutritional status before pregnancy (based on body-mass-index BMI)			Comparison between underweight and normal weight participants		Comparison between overweight and normal weight participants	
	Group	Normal BMI(18.5–25.0 kg/m^2^)	Underweight (BMI < 18.5 kg/m^2^)	Overweight (BMI ≥25.0 kg/m^2^)	RR (95% CI)	Adjusted p-value^a^	RR (95% CI)	Adjusted p-value^a^
Incidence of preterm (GA<37wk)	IFA	42/355 (11.8%)	3/32 (9.4%)	4/44 (9.1%)	1.1 (0.4 to 3.4)	0.881	0.7 (0.2 to 1.9)	0.474
	MM N	34/355 (9.6%)	0/27 (0%)	7/40 (14.9%)		0.978		0.133
	LNS	34/350 (9.7%)	2/34 (5.9%)	3/43 (7.0%)	0.8 (0.2 to 2.9)	0.681	0.8 (0.3 to 2.5)	0.762
Incidence of low birth weight (<2500 g)	IFA	41/319 (12.9%)	7/27 (25.9%)	1/36 (2.8%)	1.9 (0.9 to 4.0)	0.078	0.2 (0.03 to 1.3)	0.084
	MM N	41/310 (13.2%)	3/25 (12.0%)	7/40 (17.5%)	0.9 (0.4 to 2.3)	0.895	1.4 (0.6 to 3.1)	0.417
	LNS	36/313 (11.5%)	5/29 (17.2%)	4/37 (10.8%)	2.3 (0.9 to 5.9)	0.077	0.97 (0.4 to 2.5)	0.947
Prevalence of stunting (LAZ<-2)	IFA	56/300 (18.7%)	8/26 (30.1%)	4/32 (12.5%)	1.7 (0.8 to 3.4)	0.141	0.7 (0.3 to 1.7)	0.423
	MM N	42/302 (13.9%)	4/24 (16.7%)	6/42 (14.3%)	1.3 (0.5 to 3.7)	0.555	1.1 (0.5 to 2.4)	0.766
	LNS	42/292 (14.4%)	8/30 (26.7%)	3/34 (8.8%)	1.7 (0.9 to 3.4)	0.114	0.7 (0.2 to 2.3)	0.605
Prevalence of small for gestational age (SGA)^b^	IFA	98/319 (30.7%)	13/27 (48.2%)	5/36 (13.9%)	1.6 (1.01 to 2.4)	0.047	0.4 (0.2 to 0.97)	0.042
	MM N	88/310 (28.4%)	11/25 (44.0%)	9/40 (22.5%)	1.9 (1.2 to 3.1)	0.008	0.9 (0.5 to 1.6)	0.772
	LNS	93/313 (29.7%)	10/29 (34.5%)	8/37 (21.6%)	1.3 (0.7 to 2.1)	0.404	0.8 (0.4 to 1.4)	0.405
Prevalence of small head circumference (HCZ<-2)	IFA	20/300 (6.7%)	1/26 (3.9%)	0/32 (0%)		0.600		0.977
	MM N	8/301 (72.7%)	1/25 (4.0%)	2/42 (4.8%)	1.7 (0.3 to 12.0)	0.575	1.9 (0.4 to 9.8)	0.435
	LNS	9/293 (3.1%)	1/30 (3.3%)	1/35 (2.9%)		0.953		0.843

^a^ P-values obtained from Poisson regression models. Models without RR did not converge due to the small incidence of adverse birth outcomes, so those P-values were obtained from multiple linear regression. Models were adjusted for covariates found in bivariate analysis to be associated with the birth outcome (P<0.10). Model for preterm birth was adjusted for gestational age at enrollment, HIV status, primiparity, and site of enrollment. Model for LBW was adjusted for child sex, HIV status, maternal height, primiparity, household food insecurity score, and site of enrollment. Model for newborn stunting was adjusted for HIV status, maternal age, maternal height, primiparity, and site of enrollment. Model for SGA was adjusted for child sex, gestational age at enrollment, HIV status, maternal age, maternal height, primiparity, season of enrollment, and site of enrollment. Model for small head circumference was adjusted for maternal age, maternal height, primiparity, season of enrollment, and site of enrollment

^b^ Defined as having birth weight <10th percentile for infants of the same gestational age from INTERGROWTH-21st standard

GA: Gestational age

## Discussion

Our findings from a cohort of pregnant women in rural Malawi indicate that pre-pregnancy BMI was positively associated with birth weight and HCZ, and women with low pre-pregnancy BMI had a 60% increased risk of giving birth to stunted newborns. We further observed that average weekly gestational weight gain (WWG) was strongly associated with pregnancy duration, birth weight, LAZ, and HCZ, and women who gained inadequate weight during pregnancy were at higher risk of giving birth to newborns with LBW, SGA, and small head circumference. We did not find increased risk of adverse birth outcomes among women with high pre-pregnancy BMI or WWG above IOM recommendations. These results are of public health importance as they emphasize the need for addressing proper nutrition amongst women not only during pregnancy and in the pre-pregnancy period, but also exploring other underlying factors that may influence low WWG among pregnant women in rural Malawi.

We found few other studies that have reported associations between pre-pregnancy BMI and birth outcomes in developing countries. A 2011 meta-analysis of 78 studies showed increased risks of preterm birth and LBW among underweight women, however, only a handful of the studies were from developing countries [28]. The 4 studies from developing countries included in the analysis of preterm birth showed no increased risk among underweight compared to normal weight women [28], which is in line with our findings. Although the authors of the meta-analysis did not examine newborn stunting, the 9 studies from developing countries included in the analysis of LBW showed an increased risk of LBW among underweight women [28]. While the incidence of LBW was higher among underweight compared to overweight women in our study, the difference was not significant. The relatively low prevalence of pre-pregnancy underweight in our sample limited our statistical power to detect such a difference. We did, however, find a greater prevalence of infant stunting among underweight women. More recent studies in developing countries have shown increased risks of adverse birth outcomes among women with low pre-pregnancy BMI [5,15,29]. Additionally, a prospective cohort study of over 500,000 women in rural China indicated increased risks of preterm birth and LBW among women with low pre-pregnancy BMI [28]. However, compared to our study population, the prevalence of pre-pregnancy underweight was much higher in the Chinese population, and increased from 10.4% to 14.1% in the 3-year time period of their study [30]. Similar to the meta-analysis, they didn’t examine newborn stunting.

Our observation of no increased risk of adverse birth outcomes (preterm birth, LBW, stunting and small head circumference) among high pre-pregnancy BMI women is consistent with a retrospective study of Thai women [31]. By contrast, several other studies, as well as a systematic review, reported that high pre-pregnancy BMI (>25kg/m^2^) increased the risk of preterm delivery [7,28,32]. However, it is important to note that some of those studies used different cut off points in describing BMI categories. Additionally, some studies relied on self-reported pre-pregnancy BMI [31,33]. Thus, more research is needed to investigate associations between high pre-pregnancy maternal BMI and birth outcomes in LICs, specifically in sub-Saharan Africa.

A key finding of our study was that low average WWG is an important risk factor for adverse birth outcomes, which aligns with previous studies [15,33]. For example, in our cohort of pregnant women, low WWG was associated with most of the outcomes we assessed (LBW, SGA, and small head circumference). A recent systematic review and meta-analysis reported that women with low GWG had a 3.4 times greater risk of LBW, regardless of the maternal pre-pregnancy BMI category [16] whereas in our study the relative risk was a bit lower [RR 2.0 (95% CI), 1.2 to 3.2]. Inadequate GWG was also found to be positively associated with risks of both LBW and preterm birth in a systematic review and a large retrospective cohort of Chinese nulliparous women [13,28]. However, in these studies, excessive GWG was associated with decreased risks of preterm delivery, SGA and LBW [13,28,32], which we did not find, perhaps due to the low proportion of women with excessive GWG. Given the lack of interaction between pre-pregnancy BMI and WWG, it seems that weight gain within the IOM recommendations is beneficial to all women, regardless of their BMI at the time of conception.

We explored the associations between pre-pregnancy BMI and birth outcomes within each of the intervention groups to get a sense of whether the nutritional intervention during pregnancy helped to prevent adverse birth outcomes among women with low or high pre-pregnancy BMI. Interestingly, underweight women in the iron-folic acid and multiple micronutrient supplement groups were at greater risk of giving birth to a SGA infant than normal weight women, while there was no such association among women in the lipid-based nutrient supplement (LNS) group. LNS was the only supplement of the 3 that contained calories, protein, and essential fatty acids, which may have helped protect infants of underweight women from being SGA.

One limitation of the current study is that we used BMI, a proxy for body composition, rather than the more direct methods of assessing body composition. Another limitation was that we calculated pre-pregnancy BMI from BMI at enrolment using regression modelling, rather than directly measuring pre-pregnancy BMI. The generalizability of these findings may be limited, as those included in the study were of older age, less educated, lower socio-economic status, and had a higher prevalence of primiparity compared to those excluded from the study. Additionally, the proportions with low WWG and low BMI among excluded women were slightly higher than among those included (79.9% vs 70.2% and 5.7% vs 4.1%), which may have some impact on generalizing the findings of the present study to the larger population. The strengths of this study are that it was a prospective study with a large sample size, and highly trained study staff was used to perform study protocols.

## Conclusion

Our findings support our hypothesis that low maternal WWG is an important risk factor for adverse pregnancy outcomes. These findings highlight the need for a better understanding of the reasons behind such a high prevalence of low GWG in rural Mangochi and how to improve the situation. There was a large discrepancy between the rates of pre-pregnancy underweight (5.9%) and low average WWG (71.8%), suggesting that factors other than lack of food contributes to low WWG. Therefore, programs should aim at investigating other underlying factors such as maternal infections during pregnancy that may impair appetite. Nevertheless, linear associations between pre-pregnancy BMI and birth weight and child head circumference, and increased risk of stunting among women with low pre-pregnancy BMI suggest that the importance of adequate nutrition in the pre-pregnancy period shouldn’t be overlooked.

## Supporting information

S1 Table Dietary supplements consumed by women enrolled in the iLiNS Project. (PDF)
