# Supplementary material for: Pre-pregnancy body mass index (BMI) and maternal gestational weight gain are positively associated with birth outcomes in rural Malawi
Source: PLoS One. 2018 Oct 23;13(10):e0206035. doi: 10.1371/journal.pone.0206035 (PMC6198955; doi:10.1371/journal.pone.0206035)
Supplement: S1 Table — (PDF) [file pone.0206035.s001.pdf]

S1 Table: Dietary supplements consumed by women enrolled in the iLiNS Project

Nutrient	IFA	MMN	LNS
Ration (g/day)	1 tablet	1 tablet	20 g sachet
Total energy (kcal)	0	0	118
Protein (g)	0	0	2.6
Fat (g)	0	0	10
Linoleic acid (g)	0	0	4.59
α-Linolenic acid (g)	0	0	0.59
Vitamin A (µg RE)	0	800	800
Vitamin C (mg)	0	100	100
Vitamin B1(mg)	0	2.8	2.8
Vitamin B2 (mg)	0	2.8	2.8
Niacin (mg)	0	36	36
Folic acid (µg)	400	400	400
Pantothenic acid (mg)	0	7	7
Vitamin B6 (mg)	0	3.8	3.8
Vitamin B12 (µg)	0	5.2	5.2
Vitamin D (µg)	0	10	10
Vitamin E (mg)	0	20	20
Vitamin K (µg)	0	45	45
Iron (mg)	60	20	20
Zinc (mg)	0	30	30
Cu (mg)	0	4	4
Calcium (mg)	0	0	280
Phosphorus (mg)	0	0	190
Potassium (mg)	0	0	200
Magnesium (mg)	0	0	65
Selenium (µg)	0	130	130
Iodine (µg)	0	250	250
Manganese (mg)	0	2.6	2.6
